# Impact of COVID-19 on residency choice: A survey of New York City medical students

**DOI:** 10.1371/journal.pone.0258088

**Published:** 2021-10-06

**Authors:** Kate E. Lee, Francesca Lim, Elisabeth R. Silver, Adam S. Faye, Chin Hur

**Affiliations:** 1 Vagelos College of Physicians and Surgeons, Columbia University, New York, New York, United States of America; 2 Department of Medicine, Division of General Medicine, New York-Presbyterian/Columbia University Irving Medical Center, New York, New York, United States of America; 3 Department of Psychological Sciences, Rice University, Houston, TX, United States of America; 4 Division of Gastroenterology & Hepatology, New York University Langone Health, New York, NY, United States of America; Central South University, Xiang Ya Hospital, CHINA

## Abstract

**Objectives:**

The Coronavirus disease 2019 (COVID-19) pandemic disrupted medical student education, particularly in New York City (NYC). We aimed to assess the impact of the COVID-19 pandemic on medical students’ residency choices.

**Methods:**

The authors conducted a cross-sectional survey of medical students in all years of study at four NYC medical schools (Columbia, Cornell, NYU, and SUNY Downstate). The survey was fielded from 19 Aug 2020 to 21 Sep 2020. Survey questions included items assessing COVID-19 impact on residency choices, personal impact of COVID-19, residency/specialty choices, and factors influencing these choices.

**Results:**

A total of 2310 students received the survey, with 547 (23.7%) providing partial responses and 212 (9.2%) providing valid responses for our primary analysis. 59.0% of participants thought that COVID-19 influenced their choice of residency/specialty, with 0.9% saying the influence was to a great extent, 22.2% to some extent, and 35.8% very little. On multivariable analysis, factors that were independently associated with COVID-19 impacting residency choice included low debt ($1 to $99,999: _adj_OR 2.23, 95%CI 1.02–5.03) compared with no debt and Other race/ethnicity (_adj_OR 0.26, 95%CI 0.10–0.63) compared with White race/ethnicity. On secondary analysis of all participants answering survey items for logistic regression regardless of survey completion, direct personal impact of COVID-19 was significantly associated with COVID-19 impacting specialty choice (_adj_OR 1.90, 95%CI 1.04–3.52). Moreover, 24 students (11.6%) reported a change in their top residency choice from before to during/after COVID-19, citing concerns about frontline work, work-life balance, and risk of harm.

**Conclusions:**

Our study found that 3 in 5 (59.0%) participants felt that COVID-19 impacted their residency choice, with 11.6% of respondents explicitly changing their top specialty choice. Investigating the impact of the pandemic on medical student residency considerations is crucial to understand how medical career outlooks may change in the future.

## Introduction

The Coronavirus disease 2019 (COVID-19) has spread globally, with New York City (NYC) as the initial United States (U.S.) epicenter [1]. The pandemic halted medical student clinical rotations and moved classes online [2–5]. Fourth-year medical students graduated early, and non-internal medicine physicians performed internal medicine roles to provide pandemic support, especially in NYC which experienced the first U.S. peak of COVID-19 in April 2020 amidst a backdrop of unprepared national pandemic relief and mitigation efforts [6–9].

Given these significant disruptions, there is potential for COVID-19 to influence medical student specialty choices. However, this impact is not well understood. To date, one national survey study conducted earlier in April 2020 found that 20% of medical students thought COVID-19 would affect their specialty choice; however, this study did not identify any specialties that were favored or disfavored due to COVID-19 [10].

This study aimed to assess the impact of COVID-19 on NYC medical students’ residency choices in order to forecast the changing medical landscape as a result of the pandemic. We distributed the survey during August—September 2020, after the peak of the pandemic in NYC. We sought to characterize any impact of COVID-19 on student residency choice by identifying favored and disfavored residencies, and reasons for residency choices.

## Methods

### Participants and survey administration

We conducted a cross-sectional survey of medical students at New York City medical schools that agreed to survey distribution. Student leaders and/or deans were asked to disseminate a recruitment message containing the anonymous survey link via email or Facebook group post to medical school class groups. The message was sent 1 or 2 additional times. A total of 2310 students were reached, representing all first-, second-, third-, and fourth- year medical student (MS1, MS2, MS3, MS4) classes at Columbia (n = 605), State University of New York (SUNY) Downstate (n = 800), New York University (NYU) (n = 481), and Cornell (n = 424). The survey was fielded from 19 Aug 2020 to 21 Sep 2020, at the start of the academic year. Procedures were approved by the Institutional Review Board (IRB) of Columbia University Medical Center (Protocol IRB-AAAT1961, 9/23/2020). Participants were informed of the purposes, methods, and use of data from the research in written recruitment materials and the first page of the survey, all items were optional, and the IRB waived the need for additional informed consent.

### Measures

We assessed our main outcome of the impact of COVID-19 on medical student residency choice with the question, “To what extent has COVID-19 influenced your choice of residency/specialty?” (*not at all*, *very little*, *to some extent*, *to a great extent*). To assess degree of impact of COVID-19 on students’ lives, responses were categorized into direct personal impact (e.g., having COVID-19, having a family member or friend sick or deceased due to COVID-19, or having a family member serving as a frontline worker) and no direct personal impact (e.g., being taken out of service, having no immediate family member or friend sick with COVID-19, or volunteering or researching remotely for COVID-19 support).

To characterize COVID-19’s impact on specialty choice, participants were asked “What was your presumed residency choice BEFORE COVID-19?” with response options including residencies identified by the American Association of Medical Colleges [11]. In branched logic format, participants who responded Internal Medicine, Medicine/Pediatrics, Pediatrics, or General Surgery were asked to specify subspecialty as identified by the American College of Physicians if they planned on pursuing one [12]. Participants were then asked an open-ended question to explain their residency/specialty choice and ranking. The same questions–presumed residency choice, open-ended explanation–were asked for “during/after COVID-19”. Participants also specified any residencies they were no longer considering as a result of COVID-19 and explained in an open-ended response.

Participant characteristics included medical school, school year, age, gender, race/ethnicity, marital status, parental status, and expected debt from medical school [10, 13–28]. Participants could select multiple race/ethnicity categories; for the purpose of this analysis we categorized multiple selections with Other race/ethnicity. The full survey is available in **S1 Table**. All data were cleaned by a research analyst with no personal affiliation to any medical school before providing data to other investigators.

### Statistical analysis

For the cohort of participants who provided valid responses, participant characteristics, including personal impact of COVID-19, were summarized using frequencies and percentages. Univariable analyses using χ^2^ and Fisher’s exact tests assessed associations of participant characteristics and COVID-19 influencing residency choice using the question “To what extent has COVID-19 influenced your choice of residency/specialty?” where the response options were categorized into a binary variable (yes: to a great extent, to some extent, very little; no: not at all). Multivariable logistic regression with adjusted odds ratios (_adj_OR) and 95% confidence intervals (CI) assessed the relationship between this question and the participant characteristics. To build the multivariable model, all variables with a p-value ≤0.2 on univariable analysis were included with gender, school year, and debt included *a priori* given these variables’ importance in prior literature on medical student specialty choice [13–29].

Additional descriptive statistics were used to summarize changes in specialty and subspecialty choice (See **S2 Table** for categorizations of specialties and subspecialties). Frontline specialties were defined as those specialties who were the first to care for COVID-19 patients as part of routine clinical duties and required direct contact with patients with COVID-19 (**S2 Table**). Open-ended responses explaining why students were considering or no longer considering certain specialties were categorized and counted by theme to enable qualitative analysis in a mixed-methods approach.

As all questions were optional, as a secondary analysis, each analysis was reconducted using a larger cohort of participants who contributed partial responses to the survey. All statistical analyses were conducted using software R, v.4.0.0.

## Results

**Fig 1:**
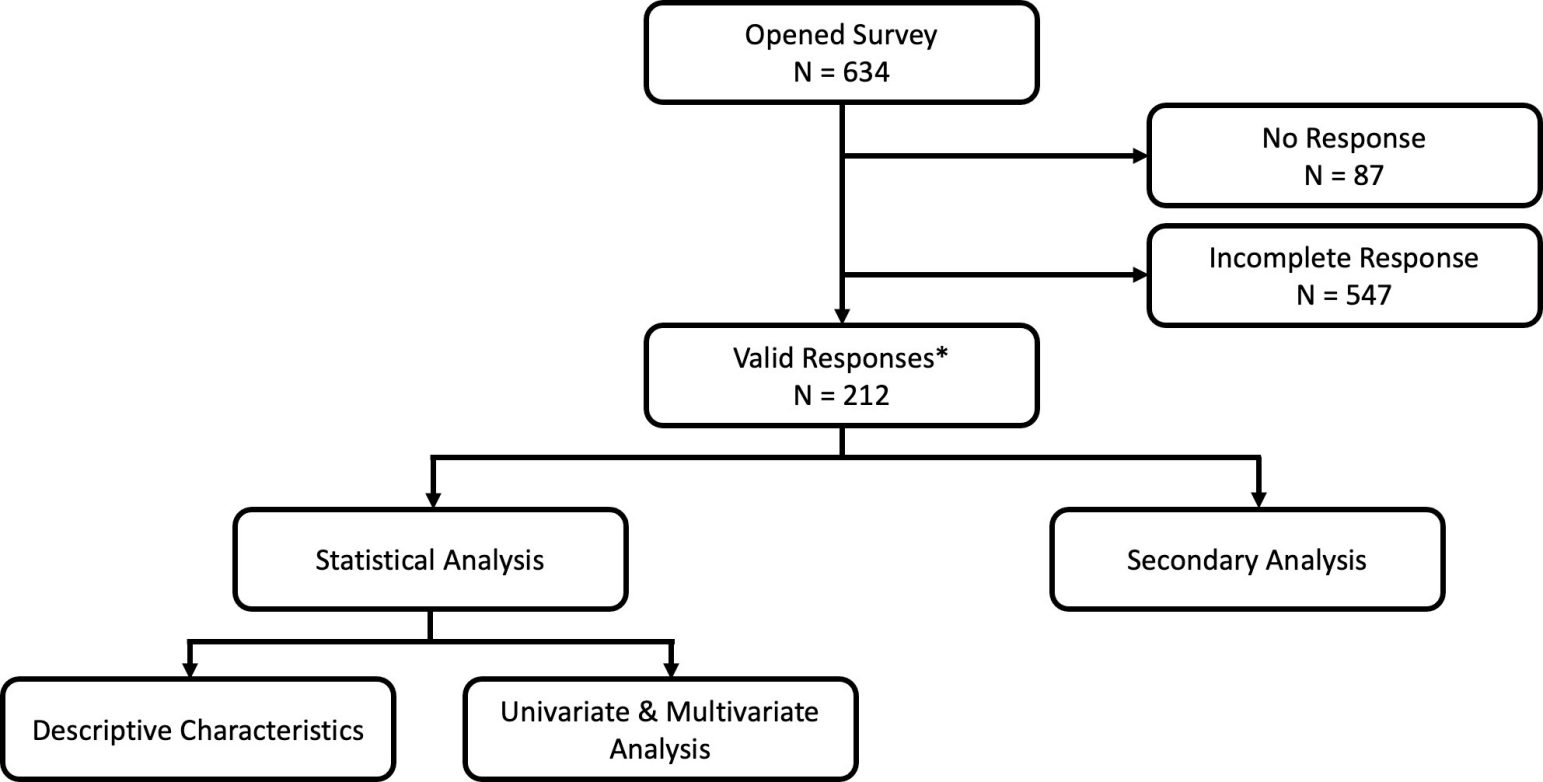
Participant flow chart. Participation by medical students in the survey. *A valid response constituted completing all demographics questions and all questions in our logistic regression.

A total of 2310 students received the survey, with 547 (23.7%) providing partial responses and 212 (9.2%) providing valid responses that were used for our primary analysis (**Fig 1**). The majority of participants were between the ages of 25 to 29 (58.0%), female (55.2%), White (54.7%), single (87.7%), and with no children (98.6%) (**Table 1**). Similar numbers of students in all medical school years participated in the survey. Students from Columbia (47.2%), SUNY Downstate (28.3%), Cornell (13.2%), and NYU (11.3%) participated. Almost one-third of participants (30.7%) reported no anticipated medical school debt, while the top one-fifth (21.7%) reported anticipated debt of $200,000 or more (24.0%). A majority (66.0%) of participants had no direct personal impact from COVID-19, while 34.0% had at least one direct personal impact. Examples of no direct personal impact included being taken out of service (78.3%) while those of direct personal impact included death of a family member (5.7%), having COVID-19 (8.5%), and having a family member with COVID-19 (26.9%) (**S3 Table**).

**Table 1:** Characteristics of 212 New York City medical student respondents.

Characteristic	N (%)
**Age**	
20–24	76 (35.8)
25–29	123 (58.0)
≥30	13 (6.1)
**Medical School**	
Columbia	100 (47.2)
Cornell	28 (13.2)
NYU	24 (11.3)
SUNY Downstate	60 (28.3)
**Medical School Year**	
MS1	49 (23.1)
MS2	50 (23.6)
MS3	43 (20.3)
MS4	58 (27.4)
Other^a^	12 (5.7)
**Gender**	
Male	91 (42.9)
Female	117 (55.2)
Other^b^	4 (1.9)
**Race/Ethnicity**	
Asian/Asian American	41 (19.3)
Black/African American	11 (5.2)
White	116 (54.7)
Hispanic/Latinx	15 (7.1)
Other, including multiracial^c^	29 (13.7)
**Marital Status**	
Single	186 (87.7)
Married	22 (10.4)
Other	4 (1.9)
**Parental Status**	
Children	3 (1.4)
No children	209 (98.6)
**Expected Debt from Medical School**	
No debt ($0)	65 (30.7)
$1 to $99,999	58 (27.4)
$100,000 to $199,999	43 (20.3)
$200,000 or more	46 (21.7)
**Personal Impact of COVID-19**	
No Direct Personal Impact	140 (66.0)
Direct Personal Impact	72 (34.0)

Abbreviations: New York University (NYU), State University of New York (SUNY), First Year Medical Student (MS1), Second Year Medical Student (MS2), Third Year Medical Student (MS3), Fourth Year Medical Student (MS4), Coronavirus disease 2019 (COVID-19).

^a^ Includes students pursuing an MD-PhD or taking a gap year.

^b^ Includes nonbinary students and those who preferred not to answer.

^c^ Includes students who preferred not to answer.

Of 212 participants who provided valid responses, 59.0% of participants thought COVID-19 influenced their choice of residency/specialty, with 0.9% saying the influence was to a great extent, 22.2% to some extent, and 35.8% very little. On univariable analysis, race/ethnicity was significantly associated with COVID-19 influencing residency choice (*p* = 0.01, **Table 2**).

**Table 2:** Univariable analysis of factors impacting residency choice During COVID-19, in 212 New York City medical students.

Characteristic	COVID-19 Influenced Residency Choice, n (%)		P Value
	No (n = 87)	Yes (n = 125)	
**Age**			0.15
20–24	32 (36.8)	44 (35.2)	
25–29	53 (60.9)	70 (56.0)	
≥30	2 (2.3)	11 (8.8)	
**Medical School**			0.62
Columbia	43 (49.4)	57 (45.6)	
Cornell	10 (11.5)	18 (14.4)	
NYU	12 (13.8)	12 (9.6)	
SUNY Downstate	22 (25.3)	38 (30.4)	
**Medical School Year**			0.72
MS1	21 (24.1)	28 (22.4)	
MS2	20 (23.0)	30 (24.0)	
MS3	14 (16.1)	29 (23.2)	
MS4	27 (31.0)	31 (24.8)	
Other^a^	5 (5.7)	7 (5.6)	
**Gender**			0.96
Male	38 (43.7)	53 (42.4)	
Female	47 (54.0)	70 (56.0)	
Other^b^	2 (2.3)	2 (1.6)	
**Race/Ethnicity**			0.01
Asian/Asian American	15 (17.2)	26 (20.8)	
Black/African American	7 (8.0)	4 (3.2)	
White	40 (46.0)	76 (60.8)	
Hispanic/Latinx	5 (5.7)	10 (8.0)	
Other, including multiracial^c^	20 (23.0)	9 (7.2)	
**Marital Status**			0.56
Single	75 (86.2)	111 (88.8)	
Married	11 (12.6)	11 (8.8)	
Other	1 (1.1)	3 (2.4)	
**Parental Status**			0.57
Children	2 (2.3)	1 (0.8)	
No children	85 (97.7)	124 (99.2)	
**Expected Debt from Medical School**			0.27
No debt ($0)	32 (36.8)	33 (26.4)	
$1 to $99,999	19 (21.8)	39 (31.2)	
$100,000 to $199,999	19 (21.8)	24 (19.2)	
$200,000 or more	17 (19.5)	29 (23.2)	
**Personal Impact of COVID-19**			0.14
No Direct Personal Impact	63 (72.4)	77 (61.6)	
Direct Personal Impact	24 (27.6)	48 (38.4)	

Abbreviations: New York University (NYU), State University of New York (SUNY), First Year Medical Student (MS1), Second Year Medical Student (MS2), Third Year Medical Student (MS3), Fourth Year Medical Student (MS4), Coronavirus disease 2019 (COVID-19).

^a^ Includes students pursuing an MD-PhD or taking a gap year.

^b^ Includes nonbinary students and those who preferred not to answer.

^c^ Includes students who preferred not to answer.

On multivariable logistic regression, characteristics associated with COVID-19 impact on residency/specialty low medical school debt compared with no debt ($1 to $99,999: _adj_OR 2.23, 95%CI 1.02–5.03) and Other race/ethnicity compared with White race/ethnicity (_adj_OR 0.26, 95%CI 0.10–0.63) (**Table 3**). As a sensitivity analysis, when medical school year was grouped into MS1 and MS2 vs. MS3 and MS4, vs. PhD or gap year, we also found this variable not significantly associated with our outcome (**S4 Table**).

**Table 3:** Multivariable logistic regression of characteristics associated with COVID-19 impacting specialty choice, in 212 New York City medical students.

Participant Characteristics	Adjusted Odds Ratio (95% CI)
**Age**	
20–24	Reference
25–29	1.31 (0.60–2.88)
≥30	4.10 (0.83–31.30)
**Medical School Year**	
MS1	Reference
MS2	1.02 (0.41–2.52)
MS3	1.12 (0.42–3.01)
MS4	0.62 (0.22–1.71)
Other^a^	0.76 (0.17–3.59)
**Gender**	
Male	Reference
Female	1.12 (0.61–2.04)
Other^b^	0.87 (0.09–9.09)
**Race/Ethnicity**	
White	Reference
Black/African American	0.31 (0.07–1.18)
Hispanic/Latinx	0.98 (0.30–3.53)
Asian	0.99 (0.45–2.33)
Other, including multiracial^c^	0.26 (0.10–0.63)
**Expected Debt from Medical School**	
No debt ($0)	Reference
$1 to $99,999	2.23 (1.02–5.03)
$100,000 to $199,999	1.30 (0.56–3.09)
$200,000 or more	1.44 (0.63–3.37)
**Personal Impact of COVID-19**	
No Direct Personal Impact	Reference
Direct Personal Impact	1.79 (0.95–3.47)

Abbreviations: Coronavirus disease 2019 (COVID-19), First Year Medical Student (MS1), Second Year Medical Student (MS2), Third Year Medical Student (MS3), Fourth Year Medical Student (MS4).

^a^ Includes students pursuing an MD-PhD or taking a gap year.

^b^ Includes nonbinary students and those who preferred not to answer.

^c^ Includes students who preferred not to answer.

As a secondary analysis, we examined all participants who provided responses to any question for demographic information (**S5 Table**), univariable analysis (**S6 Table**), and multivariable analysis (**S7 Table**). We found that of 463 participants that responded to the question assessing our main outcome of COVID-19 impact on residency choice, 59.2% of participants thought that COVID-19 influenced their choice, with 1.7% saying the influence was to a great extent, 21.4% to some extent, and 36.1% very little. On univariable analysis, medical school year and race/ethnicity were significantly associated with COVID-19 influencing residency choice (*p* = 0.01 and *p* = 0.04 respectively, **S6 Table**). On multivariable logistic regression (*n* = 243), low medical school debt and Other race/ethnicity were significantly associated with our composite outcome (**S7 Table**). Direct personal impact of COVID-19 also emerged as significantly associated with our outcome of COVID-19 impacting specialty choice (_adj_OR 1.90, 95%CI 1.04–3.52). Adjusting for participant characteristics, the effect of medical school year was no longer associated with our outcome.

In our primary cohort who provided valid responses, 24 (11.6%) indicated a change in their top specialty choice from one specialty to another (**S8 Table**). Of these 24 students, 2 (8.3%) moved towards frontline specialties while 7 (29.1%) moved away from them. A net of 5 (20.8%) students changed from Medicine to another specialty, and a net of 5 (20.8%) students changed from another specialty into Psychiatry. When we expanded our cohort to include 326 students who answered this particular survey item, we found that 47 (14.4%) students explicitly noted a change in their top specialty choice (**S8 Table**). 12 (25.5%) changed from a non-frontline specialty towards a frontline specialty, while 10 (21.2%) changed from a frontline specialty to a non-frontline specialty. A net of 7 students (14.9%) changed from Ophthalmology/Otolaryngology to another specialty, while a net of 5 students (10.6%) changed into Psychiatry. Of the students who changed their subspecialty choice within Internal Medicine, Medicine-Pediatrics, Pediatrics, or General Surgery, there were no obvious favored or disfavored specialties (**S9 Table**).

Open-ended responses revealed that students were both drawn towards and away from frontline specialties. In our cohort for primary analysis, 64 participants provided open-ended responses, of which a total of 11 (17.2%) expressed they were drawn away from frontline specialties (**S10 Table**). Specifically, 9 (14.0%) students expressed they were no longer considering Emergency Medicine, citing the exposure to dangers associated with pandemics for doctors in this field. However, 3 students (4.7%) expressed they were more drawn to frontline work due to the increased importance of Emergency Medicine during a pandemic. Additionally there were 3 students (4.7%) no longer considering surgery, with one explaining they were drawn away due to the large number of elective procedures that were cancelled in the wake of the pandemic. Some students expressed more general concerns, including increased concerns regarding work-life balance (6.3%), concern for administrative (hospital or government) support for physicians (6.3%), and fear for the safety of doctors in pandemic situations (7.8%). When we looked at all respondents who provided open-ended responses regardless of overall survey completion, concerns were similar to our main cohort.

## Discussion

Our study assessing the impact of COVID-19 on medical students’ choice of residency found that 3 in 5 NYC medical students (59.0%) reported an influence of COVID-19 on residency choice. Factors independently associated with COVID-19 impact on residency choice included low medical school debt and Other race/ethnicity. Our secondary analysis, which included all responses of students who answered the necessary items for our logistic regression found that direct personal impact of COVID-19 was also significantly associated with COVID-19 impacting specialty choice. Twenty-four (11.6%) students in our primary analysis and 47 (14.4%) students in our expanded secondary analysis indicated a change in their first residency choice since the start of the pandemic. Students had varied reasons for deciding for or against certain specialties, with some drawn towards and others away from frontline specialties.

Our finding that 59.0% of NYC medical students believed that COVID-19 impacted their residency choice is a larger percentage than has been previously reported, as Byrnes et al. found that 20% of medical students across the U.S. thought COVID-19 would affect their specialty choice prior to the peak of the pandemic in April 2020 [10]. Our findings are likely higher because NYC medical students represent a different cohort given that the city was initially the hardest-hit area in early-mid 2020 as the U.S. epicenter [7]. Pandemic relief efforts were unprepared, at times failing to provide personal protective equipment, testing, and tracing to keep healthcare workers safe while the city’s hospitals were bombarded with COVID-19 cases [8, 9]. Our study captures the considerations of NYC medical students after the initial peak in the city. In our secondary analysis including all partial survey responders, we found that personal impact of COVID-19 was independently associated with COVID-19 impacting specialty choice. This finding suggests that a student’s personal experiences throughout the pandemic may play a role in their specialty choice considerations. Our findings are in line with an international survey of residents and fellows that found that direct COVID-19 patient care as well as caring for more COVID-19 patients was independently associated with reporting burnout, another measure of pandemic effect [30].

Several previous studies published prior to the COVID-19 pandemic examined factors influencing specialty choice, mostly with null results, although a handful of studies showed associations between debt and specialty choice [13–21]. A systematic review of medical student debt on specialty choice found 21 articles showing associations between debt and choosing a higher paying specialty, though a minority (9) of articles found the opposite [13]. Likewise, in our study, we found that debt was indeed a significant variable in residency choice considerations, with low debt independently associated with COVID-19 impact on specialty choice. When we examined the debt of the 47 students who indicated change in specialty, there were no apparent tendencies of students with low or high anticipated debt to choose low- or high-paying specialties (See **S2 Table** for categorization of specialties) [13].

Currently, the impact of race/ethnicity on medical student specialty choice in general is not well studied [31, 32]. We found that Other race/ethnicity was independently associated with our outcome. When we looked into race/ethnicity of the total of 47 students who indicated that they changed specialties, 7 students indicated Other race/ethnicity of which 3 changed their choice from a non-frontline high-paying specialty to a frontline low-paying one. Further research should explore the relationship between race/ethnicity and specialty choice. Finally, although a few studies have reported gender as an important factor in choosing specialty, gender was not associated with our outcome [22–25].

The 47 total participants who indicated an explicit change in their specialty choice represent at least 2.0% of the entire 2310 individuals reached with the survey. This is a not insignificant number to explicitly note a change amidst COVID-19, especially given that many individuals who were reached did not provide even partial responses. These students were spread across all medical school years, with more students further along in their medical school education; 15 (31.9%) students were MS4, 13 (27.7%) MS3, 9 (19.9%) MS2, 9 (19.9%) MS1, and 1 (2.1%) Other. Twelve (25.5%) students moved towards frontline specialties (4 MS4, 4 MS3, 4 MS1) while 10 (21.2%) moved away from them (5 MS4, 1 MS3, 2 MS2, and 2 MS1). In our primary cohort of 212 students who completed the survey, 24 (11.6%) indicated a change in their specialty choice from one specialty to another. They were spread across MS year, as 6 (25%) were MS4, 6 (25%) MS3, 8 (33.3%) MS2, and 4 (16.7%) MS1. Two (8.3%) students, both MS1, moved towards frontline specialties while 7 (29.1%) moved away from them (3 MS4, 1 MS3, 2 MS2, and 1 MS1). The relative shift away from frontline specialties in those who provided valid responses compared to those who did not may indicate that students who completed more of the survey may have been more negatively impacted by the COVID-19 pandemic. Overall, the breakdown of MS year corroborates our finding in this study that MS year was not significantly associated with COVID-19 impacting medical student specialty choice.

Of all students who provided open-ended responses, 5 (4.4%) students wrote they were drawn towards frontline specialties while 23 (20.2%) students were drawn away from them, citing reasons such as risk and work-life balance versus importance of such work. Similarly, a survey of medical students’ specialty choices amidst an economic crisis in Spain also found wide variation in the importance that medical students attached to a controllable lifestyle and reduced work hours, with some students valuing longer than shorter workdays [33]. Conflicting reasons alongside a lack of obviously favored or disfavored specialties from our results suggest that COVID-19 will affect different students in different ways.

One of the biggest changes among all 47 participants who changed specialty considerations was a movement towards Psychiatry, as a net of 5 students (10.6%) chose Psychiatry. Students cited the prevalence of psychiatric illness in people without homes in New York and cited the importance of psychiatry in stressful times such as the pandemic (**S10 Table**). A systematic review and meta-analysis of the psychological impact of the pandemic found that risk factors for increased prevalence of anxiety and depression included high COVID-19 contraction risk and social isolation [34]. Protective factors included having sufficient medical resources, accurate information, and precautionary measures [34]. During the peak of the pandemic in NYC, there were insufficient resources, conflicting information at times, and mandatory stay at home orders, likely contributing to increased stress and anxiety [7, 8]. A study in medical trainees found that working on frontlines was associated with higher risk of developing symptoms of depression, anxiety, and acute stress disorder [35]. The psychological impact of COVID-19 has been far-reaching, and the salience of psychiatry in the pandemic may be informing medical students’ specialty considerations.

A strength of our study is that we assessed NYC medical students after the peak of the pandemic in NYC. As NYC was the first epicenter of COVID-19 in the U.S., we are among the first to assess changes in medical students’ specialty choices due to the pandemic.

Our study also has limitations. We queried individuals about their considerations “before” and “during/after” COVID-19 at one cross-sectional time point. As the pandemic continues, these considerations may also change. Additionally, our study had a low response rate, introducing response bias. As our entire study was optional and not a unique link, a participant could complete only parts of the survey or potentially complete it multiple times. As such, there were missing data for covariates, resulting in a cohort of 212 students who completed all the necessary information including demographic information, questions on COVID-19 impact personally and on specialty, that qualified for full analysis in our multivariable model. Our study may not be generalizable as NYC medical students might represent a different cohort from students in other areas of the country.

In conclusion, we found that majority (59.0%) of medical student participants report that COVID-19 influenced their residency choice albeit in different ways. Low debt and Other race/ethnicity were independent factors associated with COVID-19 impacting residency choice. It is imperative that we understand the ways medical student specialty considerations might change amidst this pandemic; as the pandemic continues to impact medical students in unprecedented ways, efforts are needed to observe how the medical landscape might shift over the following years.

## Supporting information

S1 Table Survey items, 19 Aug 2020 to 21 Sep 2020. Abbreviations: Coronavirus disease 2019 (COVID-19), First Year Medical Student (MS1), Second Year Medical Student (MS2), Third Year Medical Student (MS3), Fourth Year Medical Student (MS4)
(PDF)

S2 Table List of categories of specialties and specialties included. (PDF)

S3 Table Different types of impact of COVID-19 in participants. Abbreviations: Coronavirus disease 2019 (COVID-19). ^a^ Denominator for percentages is the number of respondents in the primary analysis. Percentages do not total to 100 because a respondent could select multiple choices. ^b^ Denominator for percentages is the number of respondents who answered this particular question as they could select more than one option. Percentages do not total to 100 because a respondent could select multiple choices. ^c^ Other direct personal impact examples include having a friend sick with COVID-19 and having a family member working on the frontline with COVID-19 patients. ^d^ Other no direct personal impact examples include remote COVID-19 related volunteering and remote COVID-19 related research.
(PDF)

S4 Table Multivariable logistic regression of characteristics associated with COVID-19 impacting specialty choice, in 212 New York City medical students, with grouped medical school year categories. Abbreviations: Coronavirus disease 2019 (COVID-19), First Year Medical Student (MS1), Second Year Medical Student (MS2), Third Year Medical Student (MS3), Fourth Year Medical Student (MS4). ^a^ Includes students pursuing an MD-PhD or taking a gap year. ^b^ Includes nonbinary students and those who preferred not to answer. ^c^ Includes students who preferred not to answer.
(PDF)

S5 Table Characteristics of 612 New York City medical student respondents. **A**bbreviations: New York University (NYU), State University of New York (SUNY), First Year Medical Student (MS1), Second Year Medical Student (MS2), Third Year Medical Student (MS3), Fourth Year Medical Student (MS4), Coronavirus disease 2019 (COVID-19). ^a^ Counts may not total to 612 due to item nonresponse. ^b^ Includes students pursuing an MD-PhD or taking a gap year. ^c^ Includes nonbinary students and those who preferred not to answer. ^d^ Includes students who preferred not to answer
(PDF)

S6 Table Univariable analysis of factors impacting residency choice during COVID-19, in 463 New York City medical students. Abbreviations: New York University (NYU), State University of New York (SUNY), First Year Medical Student (MS1), Second Year Medical Student (MS2), Third Year Medical Student (MS3), Fourth Year Medical Student (MS4), Coronavirus disease 2019 (COVID-19). ^a^ Counts may not total to specified values due to item nonresponse. ^b^ Includes students pursuing an MD-PhD or taking a gap year. ^c^ Includes nonbinary students and those who preferred not to answer. ^d^ Includes students who preferred not to answer
(PDF)

S7 Table Multivariable logistic regression of characteristics associated with COVID-19 impacting specialty choice, in 243^a^ New York City medical students. Abbreviations: Coronavirus disease 2019 (COVID-19), First Year Medical Student (MS1), Second Year Medical Student (MS2), Third Year Medical Student (MS3), Fourth Year Medical Student (MS4). ^a^ Because of missing covariates, 369 responses were not included in the model. ^b^ Includes students pursuing an MD-PhD or taking a gap year. ^c^ Includes nonbinary students and those who preferred not to answer. ^d^ Includes students who preferred not to answer
(PDF)

S8 Table Changes in specialty choice in participants, from before and after COVID-19. Abbreviations: Coronavirus disease 2019 (COVID-19), Obstetrics and Gynecology (OBGYN). ^a^ Denominator for percentages is the number of respondents in the primary analysis who answered both before and after parts of this particular survey item. The total n does not equal n = 212 of the primary analysis as our inclusion criteria did not necessitate participants to specify specialty before and after COVID-19. ^b^ Percentages in this column represent the percent change between the two counts in the Data column. ^c^ Denominator for percentages is the total number of participants who answered this particular survey item, both before and after parts, regardless of answering any other parts of the survey.
(PDF)

S9 Table Changes in internal medicine, medicine-pediatrics, pediatrics, and general surgery subspecialty choice in participants, before and after COVID-19^a^. Abbreviations: Coronavirus disease 2019 (COVID-19). ^a^ Questions about subspecialty choice were only presented to respondents who indicated an interest in Internal Medicine, Med-Peds, Pediatrics, and General Surgery. Only participants’ first choice was counted to avoid counting a participant more than once. ^b^ Denominator for percentages is the number of respondents in the primary analysis who answered both before and after parts of this particular survey item. The total n does not equal n = 212 of the primary analysis as not all participants were considering Internal Medicine, Medicine-Pediatrics, Pediatrics, and General Surgery. In addition, the primary analysis inclusion criteria did not necessitate participants to specify specialty before and after COVID-19. ^c^ Denominator for percentages is the total number of participants who answered this particular survey item, both before and after parts, regardless of answering any other parts of the survey.
(PDF)

S10 Table Reasons participants provided for considering or not considering certain residencies/specialties. Abbreviations: Coronavirus disease 2019 (COVID-19, COVID), Emergency Medicine (EM), Internal Medicine (IM), Emergency Room (ER), Intensive Care Unit (ICU). ^a^ Denominator for percentages is the total number of participants who provided open-ended responses, regardless of answering any other parts of the survey. ^b^ Denominator for percentages is the number of respondents in the primary analysis who provided open-ended responses. The total n does not equal n = 212 of the primary analysis as the primary analysis inclusion criteria did not necessitate participants to provide open-ended responses.
(PDF)

## Abbreviations

COVID-19: Coronavirus Disease 2019
NYC: New York City
U.S: United States
NYU: New York University
SUNY: State University of New York
MS1: First Year Medical Student
MS2: Second Year Medical Student
MS3: Third Year Medical Student
MS4: Fourth Year Medical Student
_adj_OR: adjusted odds ratios
CI: confidence intervals
