# Supplementary material for: Impact of COVID-19 on residency choice: A survey of New York City medical students
Source: PLoS One. 2021 Oct 6;16(10):e0258088. doi: 10.1371/journal.pone.0258088 (PMC8494369; doi:10.1371/journal.pone.0258088)
Supplement: S1 Table — Abbreviations: Coronavirus disease 2019 (COVID-19), First Year Medical Student (MS1), Second Year Medical Student (MS2), Third Year Medical Student (MS3), Fourth Year Medical Student (MS4) (PDF) [file pone.0258088.s001.pdf]

S1 Table. Survey Items, 19 Aug 2020 to 21 Sep 2020.

**Survey Item**	**Response Options**
What medical school do you attend?	Text
What year in medical school are you?	MS1<br>MS2<br>MS3<br>MS4<br>Recent graduate (currently intern)<br>Year “off”(what year did you complete? Please describe what you are doing during year off) – Text<br>Other – Text
Had you decided on a residency/specialty before Covid-19?	Yes<br>No
Have you decided on a residency/specialty as of today?	Yes<br>No
As a result of Covid-19, are there residencies/specialties that you would no longer consider specializing in?	Yes<br>No
If yes, what residencies/specialties would you no longer consider and why?<br>If no, why not?	Open ended
How much exposure to Covid-19 did you have? (You may select more than one response.)	I was taken out of service from the hospital/campus.<br>I was sick with Covid-19 myself.<br>My family member had Covid-19.<br>My friend died from Covid-19.<br>Other – Text
To what extent has Covid-19 influenced your choice of residency/specialty?	To a great extent<br>To some extent<br>Very little<br>Not at all
Please respond to this statement. Covid-19 impacted my choice of residency of specialty.	Strongly disagree<br>Disagree<br>Somewhat disagree<br>Neither agree nor disagree<br>Somewhat agree<br>Agree<br>Strongly agree
What was your presumed residency choice before Covid-19? (Drag and drop.)<br>First Choice<br>Second Choice<br>Third Choice	Anesthesiology<br>Internal Medicine<br>Child Neurology<br>Dermatology<br>Emergency Medicine<br>Family Medicine<br>Interventional Radiology<br>General Surgery<br>Medical Genetics<br>Medicine/Pediatrics<br>Neurology<br>Neurosurgery<br>Nuclear Medicine<br>Obstetrics/Gynecology<br>Ophthalmology<br>Orthopedic Surgery

	Otolaryngology<br>Pathology<br>Pediatrics<br>Physical Medicine & Rehabilitation<br>Plastic Surgery<br>Preventive Medicine<br>Psychiatry<br>Radiation Oncology<br>Radiology, Diagnostic<br>Thoracic Surgery<br>Urology<br>Vascular Surgery<br>Other – Text
If Internal Medicine/Medicine-Pediatrics/Pediatrics, were you planning on pursuing a subspecialty?	Yes<br>No<br>Undecided<br>Other – Text
If yes: What subspecialty within this residency were you interested in pursuing, if you knew?	Allergy and Immunology<br>Cardiology<br>Endocrinology, Diabetes, and Metabolism<br>Gastroenterology<br>Hematology<br>Infectious Disease<br>Nephrology<br>Oncology<br>Pulmonary Disease<br>Rheumatology<br>Other – Text
If General Surgery, were you planning on pursuing a subspecialty?	Yes<br>No<br>Undecided<br>Other
If yes: What subspecialty within this residency were you interested in pursuing, if you knew?	Breast Surgery<br>Cardiothoracic Surgery<br>Colorectal Surgery<br>Critical Care Medicine<br>Hand Surgery<br>Minimally Invasive Surgery<br>Surgical Critical Care<br>Surgical Oncology<br>Transplant Surgery<br>Vascular Surgery<br>Other – Text
Rank the factors that influenced your decision to pursue this residency/specialty before Covid-19.<br>1<br>2<br>3	Debt/finances<br>Desire for longitudinal patient care<br>Interest in global health/volunteer opportunities<br>Influential role model in residency/specialty<br>Work-life balance<br>Research Interest<br>Risk of personal harm<br>Risk of harm to family<br>Opportunity to explore my specialty of interest<br>Ability to bolter residency application<br>Other - Text
Please explain your choice and ranking.	Open ended

What is your presumed residency choice during/after Covid-19? (Drag and drop.)<br>First Choice<br>Second Choice<br>Third Choice	Anesthesiology<br>Internal Medicine<br>Child Neurology<br>Dermatology<br>Emergency Medicine<br>Family Medicine<br>Interventional Radiology<br>General Surgery<br>Medical Genetics<br>Medicine/Pediatrics<br>Neurology<br>Neurosurgery<br>Nuclear Medicine<br>Obstetrics/Gynecology<br>Ophthalmology<br>Orthopedic Surgery<br>Otolaryngology<br>Pathology<br>Pediatrics<br>Physical Medicine & Rehabilitation<br>Plastic Surgery<br>Preventive Medicine<br>Psychiatry<br>Radiation Oncology<br>Radiology, Diagnostic<br>Thoracic Surgery<br>Urology<br>Vascular Surgery<br>Other – Text
If Internal Medicine/Medicine-Pediatrics/Pediatrics, are you planning on pursuing a subspecialty?	Yes<br>No<br>Undecided<br>Other – Text
If yes: What subspecialty within this residency are you interested in pursuing, if you knew?	Allergy and Immunology<br>Cardiology<br>Endocrinology, Diabetes, and Metabolism<br>Gastroenterology<br>Hematology<br>Infectious Disease<br>Nephrology<br>Oncology<br>Pulmonary Disease<br>Rheumatology<br>Other – Text
If General Surgery, are you planning on pursuing a subspecialty?	Yes<br>No<br>Undecided<br>Other
If yes: What subspecialty within this residency are you interested in pursuing, if you knew?	Breast Surgery<br>Cardiothoracic Surgery<br>Colorectal Surgery<br>Critical Care Medicine<br>Hand Surgery<br>Minimally Invasive Surgery<br>Surgical Critical Care<br>Surgical Oncology

	Transplant Surgery<br>Vascular Surgery<br>Other – Text
Indicate the top three (3) factors that are influencing your decision to pursue this residency/specialty during/after Covid-19?<br>1<br>2<br>3	Debt/finances<br>Desire for longitudinal patient care<br>Interest in global health/volunteer opportunities<br>Influential role model in residency/specialty<br>Work-life balance<br>Research Interest<br>Risk of personal harm<br>Risk of harm to family<br>Opportunity to explore my specialty of interest<br>Ability to bolter residency application<br>Other - Text
Please explain your choice and ranking.	Open ended
Age	Dropdown
What is your gender?	Female<br>Male<br>Nonbinary<br>Prefer not to say<br>Other – Text
How do you identify your race and/or ethnicity?	Asian/Asian American<br>Black/African American<br>Caucasian<br>Hispanic/Latinx<br>Native American<br>Pacific Islander<br>Prefer not to answer<br>Other – Text
Marital Status	Single<br>Married<br>Widowed<br>Divorced<br>Separated<br>Other – Text
Do you have children?	Yes<br>No<br>Expecting a child soon<br>Other – Text
What is your expected debt from medical school?	No debt ($0)<br>$1 to $49,999<br>$50,000 to $99,999<br>$100,000 to $149,999<br>$150,000 to $199,999<br>$200,000 to $299,999<br>$300,000 to $399,999<br>$400,000 or more
Any comments/questions/concerns?	Open ended

Abbreviations: Coronavirus disease 2019 (COVID-19), First Year Medical Student (MS1), Second Year Medical Student (MS2), Third Year Medical Student (MS3), Fourth Year Medical Student (MS4)
