# Supplementary material for: Impact of COVID-19 on residency choice: A survey of New York City medical students
Source: PLoS One. 2021 Oct 6;16(10):e0258088. doi: 10.1371/journal.pone.0258088 (PMC8494369; doi:10.1371/journal.pone.0258088)
Supplement: S2 Table — (PDF) [file pone.0258088.s002.pdf]

S2 Table. List of Categories of Specialties and Specialties Included.

Category	Specialties
**Medicine**	Internal Medicine, Medicine-Pediatrics, Pediatrics, Geriatrics, Family Medicine, Social Medicine, Physical Medicine and Rehabilitation, Child Neurology, Neurology, Palliative Care, Medical Oncology, Pathology
**Emergency Medicine**	Emergency Medicine
**Psychiatry**	Psychiatry
**Dermatology**	Dermatology
**Radiology**	Radiology, Nuclear Medicine, Radiation Oncology
**Otolaryngology/Ophthalmology**	Otolaryngology, Ophthalmology
**Surgery**	Neurosurgery, Plastic Surgery, Thoracic Surgery, General Surgery, Orthopedic Surgery
**Obstetrics and Gynecology/Urology**	Obstetrics and Gynecology, Urology
**Anesthesiology**	Anesthesiology
**Not Applicable/Undecided**	Not Applicable, Undecided
**Frontline**	Anesthesiology, Emergency Medicine, Internal Medicine, Pediatrics, Family Medicine
**Non-Frontline**	Dermatology, Obstetrics and Gynecology, Urology, Ophthalmology, Otolaryngology, Pathology, Psychiatry, Radiology, Neurosurgery, Plastic Surgery, Thoracic Surgery, General Surgery, Orthopedic Surgery
**Low-Paying**	Emergency Medicine, Internal Medicine, Family Medicine, Pediatrics, Medicine-Pediatrics, Pathology, Psychiatry, Obstetrics and Gynecology
**High-Paying**	Dermatology, Anesthesiology, Ophthalmology, Otolaryngology, Urology, Radiology, Neurosurgery, Plastic Surgery, Thoracic Surgery, General Surgery, Orthopedic Surgery
