# Supplementary material for: Impact of COVID-19 on residency choice: A survey of New York City medical students
Source: PLoS One. 2021 Oct 6;16(10):e0258088. doi: 10.1371/journal.pone.0258088 (PMC8494369; doi:10.1371/journal.pone.0258088)
Supplement: S3 Table — Abbreviations: Coronavirus disease 2019 (COVID-19). a Denominator for percentages is the number of respondents in the primary analysis. Percentages do not total to 100 because a respondent could select multiple choices. b Denominator for percentages is the number of respondents who answered this particular question as they could select more than one option. Percentages do not total to 100 because a respondent could select multiple choices. c Other direct personal impact examples include having a friend sick with COVID-19 and having a family member working on the frontline with COVID-19 patients. d Other no direct personal impact examples include remote COVID-19 related volunteering and remote COVID-19 related research. (PDF) [file pone.0258088.s003.pdf]

S3 Table. Different Types of Impact of COVID-19 in Participants.

Impact Type	Data, n=212 (%)[a]	Data, n=425 (%)[b]
**Direct Personal Impact**		
Family member died	12 (5.7)	20 (4.7)
Friend died	0 (0)	10 (2.4)
I was sick with COVID-19	18 (8.5)	41 (9.7)
Family member was sick with COVID-19	57 (26.9)	95 (22.4)
Other direct[c]	1 (0.5)	3 (0.7)
**No Direct Personal Impact**		
Taken out of service	166 (78.3)	312 (73.4)
Other no direct personal impact[d]	28 (13.2)	52 (12.2)

Abbreviations: Coronavirus disease 2019 (COVID-19)

[a] Denominator for percentages is the number of respondents in the primary analysis. Percentages do not total to 100 because a respondent could select multiple choices.

[b] Denominator for percentages is the number of respondents who answered this particular question as they could select more than one option. Percentages do not total to 100 because a respondent could select multiple choices.

[c] Other direct personal impact examples include having a friend sick with COVID-19 and having a family member working on the frontline with COVID-19 patients.

[d] Other no direct personal impact examples include remote COVID-19 related volunteering and remote COVID-19 related research.
