# Supplementary material for: Impact of COVID-19 on residency choice: A survey of New York City medical students
Source: PLoS One. 2021 Oct 6;16(10):e0258088. doi: 10.1371/journal.pone.0258088 (PMC8494369; doi:10.1371/journal.pone.0258088)
Supplement: S4 Table — Abbreviations: Coronavirus disease 2019 (COVID-19), First Year Medical Student (MS1), Second Year Medical Student (MS2), Third Year Medical Student (MS3), Fourth Year Medical Student (MS4). a Includes students pursuing an MD-PhD or taking a gap year. b Includes nonbinary students and those who preferred not to answer. c Includes students who preferred not to answer. (PDF) [file pone.0258088.s004.pdf]

S4 Table. Multivariable Logistic Regression of Characteristics Associated with COVID-19 Impacting Specialty Choice, in 212 New York City Medical Students, with Grouped Medical School Year Categories.

Participant Characteristics	Adjusted Odds Ratio (95% CI)
**Age**	
20-24	Reference
25-29	1.15 (0.43-2.29)
≥30	3.68 (0.77-27.42)
**Medical School Year**	
MS1 + MS2	Reference
MS3 + MS4	0.84 (0.41-1.69)
Other[a]	0.83 (0.21-3.45)
**Gender**	
Male	Reference
Female	1.11 (0.61-2.03)
Other[b]	0.94 (0.09-10.03)
**Race/Ethnicity**	
White	Reference
Black/African American	0.29 (0.07-1.09)
Hispanic/Latinx	0.98 (0.31-3.49)
Asian	1.01 (0.46-2.26)
Other, including multiracial[c]	0.25 (0.10-0.62)
**Expected Debt from Medical School**	
No debt ($0)	Reference
$1 to $99,999	2.12 (0.97-4.73)
$100,000 to $199,999	1.27 (0.55-2.97)
$200,000 or more	1.43 (0.63-3.33)
**Personal Impact of COVID-19**	
No Direct Personal Impact	Reference
Direct Personal Impact	1.81 (0.96-3.48)

Abbreviations: Coronavirus disease 2019 (COVID-19), First Year Medical Student (MS1), Second Year Medical Student (MS2), Third Year Medical Student (MS3), Fourth Year Medical Student (MS4)

[a] Includes students pursuing an MD-PhD or taking a gap year

[b] Includes nonbinary students and those who preferred not to answer

[c] Includes students who preferred not to answer
