# Supplementary material for: Impact of COVID-19 on residency choice: A survey of New York City medical students
Source: PLoS One. 2021 Oct 6;16(10):e0258088. doi: 10.1371/journal.pone.0258088 (PMC8494369; doi:10.1371/journal.pone.0258088)
Supplement: S5 Table — Abbreviations: New York University (NYU), State University of New York (SUNY), First Year Medical Student (MS1), Second Year Medical Student (MS2), Third Year Medical Student (MS3), Fourth Year Medical Student (MS4), Coronavirus disease 2019 (COVID-19). a Counts may not total to 612 due to item nonresponse. b Includes students pursuing an MD-PhD or taking a gap year. c Includes nonbinary students and those who preferred not to answer. d Includes students who preferred not to answer (PDF) [file pone.0258088.s005.pdf]

S5 Table. Characteristics of 612 New York City Medical Student Respondents.

Characteristic	N[a] (%)
**Age**	
20-24	99 (36.5)
25-29	152 (56.1)
≥30	20 (7.4)
**Medical School**	
Columbia	192 (44.1)
Cornell	58 (13.3)
NYU	44 (10.1)
SUNY Downstate	141 (32.4)
**Medical School Year**	
MS1	165 (31.5)
MS2	109 (20.8)
MS3	97 (18.5)
MS4	128 (24.5)
Other[b]	24 (4.6)
**Gender**	
Male	141 (42.5)
Female	185 (55.7)
Other[c]	6 (1.8)
**Race/Ethnicity**	
Asian/Asian American	62 (18.7)
Black/African American	18 (5.4)
White	188 (56.6)
Hispanic/Latinx	20 (6.0)
Other, including multiracial[d]	44 (13.3)
**Marital Status**	
Single	292 (89.3)
Married	29 (8.9)
Other	6 (1.8)
**Parental Status**	
Children	5 (1.5)
No children	326 (98.2)
Expecting a child soon	1 (0.3)
**Expected Debt from Medical School**	
No debt ($0)	96 (29.5)
$1 to $99,999	85 (26.2)
$100,000 to $199,999	66 (20.3)
$200,000 or more	78 (24.0)
**Personal Impact of COVID-19**	
No Direct Personal Impact	292 (68.7)
Direct Personal Impact	133 (31.3)

Abbreviations: New York University (NYU), State University of New York (SUNY), First Year Medical Student (MS1), Second Year Medical Student (MS2), Third Year Medical Student (MS3), Fourth Year Medical Student (MS4), Coronavirus disease 2019 (COVID-19)

[a] Counts may not total to 612 due to item nonresponse

[b] Includes students pursuing an MD-PhD or taking a gap year

[c] Includes nonbinary students and those who preferred not to answer

[d] Includes students who preferred not to answer
