# Supplementary material for: Impact of COVID-19 on residency choice: A survey of New York City medical students
Source: PLoS One. 2021 Oct 6;16(10):e0258088. doi: 10.1371/journal.pone.0258088 (PMC8494369; doi:10.1371/journal.pone.0258088)
Supplement: S6 Table — Abbreviations: New York University (NYU), State University of New York (SUNY), First Year Medical Student (MS1), Second Year Medical Student (MS2), Third Year Medical Student (MS3), Fourth Year Medical Student (MS4), Coronavirus disease 2019 (COVID-19). a Counts may not total to specified values due to item nonresponse. b Includes students pursuing an MD-PhD or taking a gap year. c Includes nonbinary students and those who preferred not to answer. d Includes students who preferred not to answer (PDF) [file pone.0258088.s006.pdf]

S6 Table. Univariable Analysis of Factors Impacting Residency Choice During COVID-19, in 463 New York City Medical Students.

Characteristic	COVID-19 Influenced Residency Choice, n (%)[a]		P Value
	No (n=189)	Yes (n=274)	
**Age**			0.12
20-24	42 (37.2)	57 (36.1)	
25-29	67 (59.3)	85 (53.8)	
≥30	4 (3.5)	16 (10.1)	
**Medical School**			0.92
Columbia	72 (45.9)	104 (44.3)	
Cornell	19 (12.1)	32 (13.6)	
NYU	17 (10.8)	22 (9.4)	
SUNY Downstate	49 (31.2)	77 (32.8)	
**Medical School Year**			0.01
MS1	61 (32.4)	78 (28.6)	
MS2	40 (21.3)	59 (21.6)	
MS3	21 (11.2)	64 (23.4)	
MS4	57 (30.3)	59 (21.6)	
Other[b]	9 (4.8)	13 (4.8)	
**Gender**			0.20
Male	63 (46.0)	78 (40.0)	
Female	70 (51.1)	115 (59.0)	
Other[c]	4 (3.0)	2 (1.0)	
**Race/Ethnicity**			0.04
Asian/Asian American	23 (16.9)	39 (19.9)	
Black/African American	9 (6.6)	9 (4.6)	
White	69 (50.7)	119 (60.7)	
Hispanic/Latinx	8 (5.9)	12 (6.1)	
Other, including multiracial[d]	27 (19.9)	17 (8.7)	
**Marital Status**			0.74
Single	120 (88.2)	172 (90.1)	
Married	14 (10.3)	15 (7.9)	
Other	2 (1.5)	4 (2.1)	
**Parental Status**			0.36
Children	1 (0.7)	4 (2.0)	
No children	134 (98.5)	192 (98.0)	
Expecting a child soon	1 (0.7)	0 (0.0)	
**Expected Debt from Medical School**			0.07
No debt ($0)	49 (37.1)	47 (24.4)	
$1 to $99,999	32 (24.2)	53 (27.5)	
$100,000 to $199,999	26 (19.7)	40 (20.7)	
$200,000 or more	25 (18.9)	53 (27.5)	
**Personal Impact of COVID-19**			0.06
No Direct Personal Impact	125 (74.4)	162 (65.1)	
Direct Personal Impact	43 (25.6)	87 (34.9)	

Abbreviations: New York University (NYU), State University of New York (SUNY), First Year Medical Student (MS1), Second Year Medical Student (MS2), Third Year Medical Student (MS3), Fourth Year Medical Student (MS4), Coronavirus disease 2019 (COVID-19)

[a] Counts may not total to specified values due to item nonresponse

[b] Includes students pursuing an MD-PhD or taking a gap year

[c] Includes nonbinary students and those who preferred not to answer

[d] Includes students who preferred not to answer
