# Supplementary material for: Impact of COVID-19 on residency choice: A survey of New York City medical students
Source: PLoS One. 2021 Oct 6;16(10):e0258088. doi: 10.1371/journal.pone.0258088 (PMC8494369; doi:10.1371/journal.pone.0258088)
Supplement: S7 Table — Abbreviations: Coronavirus disease 2019 (COVID-19), First Year Medical Student (MS1), Second Year Medical Student (MS2), Third Year Medical Student (MS3), Fourth Year Medical Student (MS4). a Because of missing covariates, 369 responses were not included in the model. b Includes students pursuing an MD-PhD or taking a gap year. c Includes nonbinary students and those who preferred not to answer. d Includes students who preferred not to answer (PDF) [file pone.0258088.s007.pdf]

S7 Table. Multivariable Logistic Regression of Characteristics Associated with COVID-19 Impacting Specialty Choice, in 243[a] New York City Medical Students.

Participant Characteristics	Adjusted Odds Ratio (95% CI)
**Age**	
20-24	Reference
25-29	1.20 (0.57-2.54)
≥30	5.26 (1.11-39.36)
**Medical School Year**	
MS1	Reference
MS2	1.11 (0.47-2.63)
MS3	1.59 (0.63-4.09)
MS4	0.69 (0.26-1.80)
Other[b]	0.71 (0.18-2.79)
**Gender**	
Male	Reference
Female	1.16 (0.66-2.05)
Other[c]	0.90 (0.09-9.34)
**Race/Ethnicity**	
White	Reference
Black/African American	0.37 (0.10-1.26)
Hispanic/Latinx	1.00 (0.30-3.64)
Asian	1.07 (0.49-2.36)
Other, including multiracial[d]	0.30 (0.13-0.69)
**Expected Debt from Medical School**	
No debt ($0)	Reference
$1 to $99,999	2.61 (1.24-5.63)
$100,000 to $199,999	1.39 (0.61-3.22)
$200,000 or more	1.97 (0.92-4.30)
**Personal Impact of COVID-19**	
No Direct Personal Impact	Reference
Direct Personal Impact	1.90 (1.04-3.52)

Abbreviations: Coronavirus disease 2019 (COVID-19), First Year Medical Student (MS1), Second Year Medical Student (MS2), Third Year Medical Student (MS3), Fourth Year Medical Student (MS4)

[a] Because of missing covariates, 369 responses were not included in the model

[b] Includes students pursuing an MD-PhD or taking a gap year

[c] Includes nonbinary students and those who preferred not to answer

[d] Includes students who preferred not to answer
