# Supplementary material for: Impact of COVID-19 on residency choice: A survey of New York City medical students
Source: PLoS One. 2021 Oct 6;16(10):e0258088. doi: 10.1371/journal.pone.0258088 (PMC8494369; doi:10.1371/journal.pone.0258088)
Supplement: S8 Table — Abbreviations: Coronavirus disease 2019 (COVID-19), Obstetrics and Gynecology (OBGYN). a Denominator for percentages is the number of respondents in the primary analysis who answered both before and after parts of this particular survey item. The total n does not equal n = 212 of the primary analysis as our inclusion criteria did not necessitate participants to specify specialty before and after COVID-19. b Percentages in this column represent the percent change between the two counts in the Data column. c Denominator for percentages is the total number of participants who answered this particular survey item, both before and after parts, regardless of answering any other parts of the survey. (PDF) [file pone.0258088.s008.pdf]

S8 Table. Changes in Specialty Choice in Participants, from Before and After COVID-19.

Data for primary analysis, n=207 (%)[a]			
**Specialty**	**Before COVID-19**	**After COVID-19**	**Net Change (n = 24)[b]**
Anesthesiology	12 (5.8)	12 (5.8)	0 (0)
Dermatology	11 (5.3)	12 (5.8)	+1 (9.1)
Emergency Medicine	14 (6.8)	14 (6.8)	0 (0)
Medicine	83 (40.1)	78 (37.7)	-5 (-6.0)
OBGYN/Urology	16 (7.7)	17 (8.2)	+1 (6.2)
Ophthalmology/Otolaryngology	19 (9.2)	16 (7.7)	-3 (-15.8)
Pathology	0 (0)	0 (0)	0 (0)
Psychiatry	13 (6.3)	18 (8.7)	+5 (38.5)
Radiology	6 (2.9)	7 (3.4)	+1 (16.7)
Surgery	33 (15.9)	33 (15.9)	0 (0)
**Data for all participants who answered this particular question, n=326 (%)[c]**			
**Specialty**	**Before COVID-19**	**After COVID-19**	**Net Change (n = 47)[b]**
Anesthesiology	14 (4.3)	15 (4.6)	+1 (7.1)
Dermatology	12 (3.7)	14 (4.3)	+2 (16.7)
Emergency Medicine	24 (7.4)	26 (8.0)	+2 (8.3)
Medicine	128 (39.4)	127 (39.1)	-1 (-0.8)
OBGYN/Urology	28 (8.6)	29 (8.9)	+1 (3.6)
Ophthalmology/Otolaryngology	25 (7.7)	18 (5.5)	-7 (-28.0)
Pathology	2 (0.6)	2 (0.6)	0 (0.0)
Psychiatry	20 (6.2)	25 (7.7)	+5 (25.0)
Radiology	11 (3.4)	12 (3.7)	+1 (9.1)
Surgery	61 (18.8)	57 (17.5)	-4 (-6.6)

Abbreviations: Coronavirus disease 2019 (COVID-19), Obstetrics and Gynecology (OBGYN)

[a] Denominator for percentages is the number of respondents in the primary analysis who answered both before and after parts of this particular survey item. The total n does not equal n=212 of the primary analysis as our inclusion criteria did not necessitate participants to specify specialty before and after COVID-19.

[b] Percentages in this column represent the percent change between the two counts in the Data column.

[c] Denominator for percentages is the total number of participants who answered this particular survey item, both before and after parts, regardless of answering any other parts of the survey.
