# Supplementary material for: Impact of COVID-19 on residency choice: A survey of New York City medical students
Source: PLoS One. 2021 Oct 6;16(10):e0258088. doi: 10.1371/journal.pone.0258088 (PMC8494369; doi:10.1371/journal.pone.0258088)
Supplement: S9 Table — Abbreviations: Coronavirus disease 2019 (COVID-19). a Questions about subspecialty choice were only presented to respondents who indicated an interest in Internal Medicine, Med-Peds, Pediatrics, and General Surgery. Only participants’ first choice was counted to avoid counting a participant more than once. b Denominator for percentages is the number of respondents in the primary analysis who answered both before and after parts of this particular survey item. The total n does not equal n = 212 of the primary analysis as not all participants were considering Internal Medicine, Medicine-Pediatrics, Pediatrics, and General Surgery. In addition, the primary analysis inclusion criteria did not necessitate participants to specify specialty before and after COVID-19. c Denominator for percentages is the total number of participants who answered this particular survey item, both before and after parts, regardless of answering any other parts of the survey. (PDF) [file pone.0258088.s009.pdf]

S9 Table. Changes in Internal Medicine, Medicine-Pediatrics, Pediatrics, and General Surgery Subspecialty Choice in Participants, Before and After COVID-19[a].

**Data for primary analysis, n=97 (%)[b]**			
**Subspecialty**	**Before COVID-19**	**After COVID-19**	**Net Change, n (%)**
Cardiology	14 (17.3)	12 (17.4)	-2 (-14.3)
Endocrinology, Diabetes, and Metabolism	1 (1.2)	2 (2.9)	1 (100.0)
Gastroenterology	11 (13.6)	8 (11.6)	-3 (-27.3)
Hematology	1 (1.2)	1 (1.4)	0 (0)
Infectious Disease	3 (3.7)	3 (4.3)	0 (0)
Nephrology	0 (0)	0 (0)	0 (0)
Oncology	16 (19.8)	13 (18.8)	-3 (-18.8)
Other	17 (21.0)	14 (20.3)	-3 (-17.6)
Pulmonary Disease	2 (2.5)	2 (2.9)	0 (0)
Rheumatology	1 (1.2)	1 (1.4)	0 (0)
Cardiothoracic Surgery	4 (4.9)	3 (4.3)	-1 (-25)
Critical Care Medicine	3 (3.7)	2 (2.9)	-1 (-33.3)
Hand Surgery	2 (2.5)	1 (1.4)	-1 (-50.0)
Minimally Invasive Surgery	1 (1.2)	1 (1.4)	0 (0)
Surgical Critical Care	2 (2.5)	3 (4.3)	1 (50.0)
Surgical Oncology	2 (2.5)	2 (2.9)	0 (0)
Transplant Surgery	0 (0)	0 (0)	0 (0)
Vascular Surgery	1 (1.2)	1 (1.4)	0 (0)
Allergy & Immunology	0 (0)	0 (0)	0 (0)
Breast Surgery	0 (0)	0 (0)	0 (0)
**Data for all participants who answered this particular question, n=156 (%)[c]**			
**Subspecialty**	**Before COVID-19**	**After COVID-19**	**Net Change, n (%)**
Cardiology	22 (14.7)	23 (16.7)	1 (4.5)
Endocrinology, Diabetes, and Metabolism	3 (2.0)	3 (2.2)	0 (0.0)
Gastroenterology	17 (11.3)	14 (10.1)	-3 (-17.6)
Hematology	7 (4.7)	7 (5.1)	0 (0.0)
Infectious Disease	10 (6.7)	8 (5.8)	-2 (-20.0)
Nephrology	2 (1.3)	1 (0.7)	-1 (-50.0)
Oncology	26 (17.3)	24 (17.4)	-2 (-7.7)
Other	29 (19.3)	25 (18.1)	-4 (-13.8)
Pulmonary Disease	4 (2.7)	4 (2.9)	0 (0.0)
Rheumatology	2 (1.3)	2 (1.4)	0 (0.0)
Cardiothoracic Surgery	9 (6.0)	8 (5.8)	-1 (-11.1)
Critical Care Medicine	3 (2.0)	3 (2.2)	0 (0.0)
Hand Surgery	3 (2.0)	2 (1.4)	-1 (-33.3)
Minimally Invasive Surgery	1 (0.7)	1 (0.7)	0 (0.0)
Surgical Critical Care	3 (2.0)	5 (3.6)	2 (66.7)
Surgical Oncology	3 (2.0)	3 (2.2)	0 (0.0)
Transplant Surgery	3 (2.0)	2 (1.4)	-1 (-33.3)
Vascular Surgery	1 (0.7)	1 (0.7)	0 (0.0)
Allergy & Immunology	2 (1.3)	1 (0.7)	-1 (-50.0)
Breast Surgery	0 (0.0)	1 (0.7)	1 (NA)

Abbreviations: Coronavirus disease 2019 (COVID-19)

[a] Questions about subspecialty choice were only presented to respondents who indicated an interest in Internal Medicine, Med-Peds, Pediatrics, and General Surgery. Only participants' first choice was counted to avoid counting a participant more than once.

[b] Denominator for percentages is the number of respondents in the primary analysis who answered both before and after parts of this particular survey item. The total n does not equal n=212 of the primary

analysis as not all participants were considering Internal Medicine, Medicine-Pediatrics, Pediatrics, and General Surgery. In addition, the primary analysis inclusion criteria did not necessitate participants to specify specialty before and after COVID-19.

[c] Denominator for percentages is the total number of participants who answered this particular survey item, both before and after parts, regardless of answering any other parts of the survey.
