# Supplementary material for: Impact of COVID-19 on residency choice: A survey of New York City medical students
Source: PLoS One. 2021 Oct 6;16(10):e0258088. doi: 10.1371/journal.pone.0258088 (PMC8494369; doi:10.1371/journal.pone.0258088)
Supplement: S10 Table — Abbreviations: Coronavirus disease 2019 (COVID-19, COVID), Emergency Medicine (EM), Internal Medicine (IM), Emergency Room (ER), Intensive Care Unit (ICU). a Denominator for percentages is the total number of participants who provided open-ended responses, regardless of answering any other parts of the survey. b Denominator for percentages is the number of respondents in the primary analysis who provided open-ended responses. The total n does not equal n = 212 of the primary analysis as the primary analysis inclusion criteria did not necessitate participants to provide open-ended responses. (PDF) [file pone.0258088.s010.pdf]

S10 Table. Reasons Participants Provided for Considering or Not Considering Certain Residencies/Specialties.

**Data for participants in primary analysis**	
**Theme and Illustrative Quotes**	**Counts**
**Reasons for Not Considering Certain Residencies/Specialties, n=64[a]**	
No Change in Specialty Consideration	31
“Was never interested in the specialties that were greatly impacted by Covid” [MS1]	
“I see covid as a normal part of medicine – there will always be risks” [MS3]	
No Longer Considering Frontline Work	11
EM/IM	9
“[…] doctors in this field are highly susceptible to the dangers of something like covid” [MS2]	
Anesthesiology	1
“[…] endlessly intubating people in critical care scenarios” [MS3]	
No Longer Considering Surgery	3
“[…] due to the vast amount of elective procedures” [MS3]	
“[…] they [surgeries] were shut down during these months” [MS3]	
More Drawn to Frontline Work	3
“[…] I am more drawn to infectious disease/ER” [MS2]	
Lack of Interest in Telehealth	1
“I don’t want to do telehealth my whole life.” [MS2]	
Inability to Explore Residency/Specialty	7
“[…] no chance for away rotations” [MS4]	
“[…] no time to gain exposure in this field” [MS4]	
Concerns About Work-Life Balance	4
“Want to be able to spend time with family” [MS3]	
Concerns About Administrative Support	4
“[…] pitfalls of the pandemic have to due with structural issues (hospital administration and government failings)” [MS4]	
“[…] the field of medicine […] has shown its lack of care of the workers” [MS2]	
Concerns About Fear and Safety	5
“lack of PPE and care […] our lives seem to be dispensable” [MS2]	
**Reasons for Considering Certain Residencies/Specialties in COVID-19, n=36[a]**	
Concerns about Burnout	1
“Seeing many people burn out in a specialty I was interested in in the midst of the pandemic was also enlightening as I realized how even in a specialty where you ‘leave work at work’ you still end up taking it home and it can literally kill you.” [MS4]	
Concerns about Work-life Balance	10
“I still aim for a productive career but know that having a good support system means more than anything.” [MS3]	
“Work life balance is key” [MS2]	
Interest or Greater Interest in Certain Specialties	6
“Taking a global health class during COVID […] solidified my decision to go into med peds” [MS4]	
“COVID-19 has only reinforced my desire to pursue psychiatry. In New York and across the country the prevalence of untreated mental illness among the homeless is particularly problematic in the setting of a global pandemic.” [Other]	
“The pandemic highlighted the value of the work of psychiatry for me. While other specialties flagged in terms of demand, psychiatry seemed to become more essential than ever.” [MS4]	
Interest in Health Systems	1
“More concerned with opportunity to pursue health equity and systems level change in addition to patient care in light of COVID-19” [MS2]	

Data for all participants who provided open-ended responses	
**Theme and Illustrative Quotes**	**Counts**
**Reasons for Not Considering Certain Residencies/Specialties, n=114[b]**	
No Change in Specialty Consideration	57
“Was never interested in the specialties that were greatly impacted by Covid” [MS1]	
“I see covid as a normal part of medicine – there will always be risks” [MS3]	
No Longer Considering Frontline Work	23
EM/IM	19
“[…] doctors in this field are highly susceptible to the dangers of something like covid” [MS2]	
“The EM doctors, especially residents, on the frontlines were treated poorly” [MS1]	
Infectious Disease	3
“lots of risk to personal health” [MS2]	
“they are not as well compensated as some other fields” [MS2]	
Anesthesiology	2
“[…] didn’t realize how much ICU work anesthesiologists did” [MS4]	
“[…] endlessly intubating people in critical care scenarios” [MS3]	
No Longer Considering Surgery	6
“[…] due to the vast amount of elective procedures” [MS3]	
“[…] they [surgeries] were shut down during these months” [MS3]	
More Drawn to Frontline Work	5
“[…] I am more drawn to infectious disease/ER” [MS2]	
“[…] COVID has heightened my awareness of what goes into EM/intensivist medicine and how important that is” [MS2]	
Lack of Interest in Telehealth	3
“I don’t want to do telehealth my whole life.” [MS2]	
“I don’t want to specialize in a field that will be mostly remote” [MS1]	
Inability to Explore Residency/Specialty	13
“[…] no chance for away rotations” [MS4]	
“[…] no time to gain exposure in this field” [MS4]	
Concerns About Work-Life Balance	7
“Want to be able to spend time with family” [MS3]	
“[…] COVID-19 has made me more aware of what I need to do to maintain my well-being” [MS2]	
Concerns About Administrative Support	7
“[…] pitfalls of the pandemic have to due with structural issues (hospital administration and government failings)” [MS4]	
“[…] the field of medicine […] has shown its lack of care of the workers” [MS2]	
Concerns About Fear and Safety	8
“My greatest concern is safety.” [MS1]	
“lack of PPE and care […] our lives seem to be dispensable” [MS2]	
**Reasons for Considering Certain Residencies/Specialties in COVID-19, n=51[b]**	
Concerns about Burnout	2
“Seeing many people burn out in a specialty I was interested in in the midst of the pandemic was also enlightening as I realized how even in a specialty where you ‘leave work at work’ you still end up taking it home and it can literally kill you.” [MS4]	
“I think that COVID made me more acutely aware of the risk of burnout, so I am considering how I might reduce that risk with my specialty choice.” [MS1]	
Concerns about Work-life Balance	4
“Ever since COVID/quarantine, I have been prioritizing my mental well-being and being able to spend time with friends and family.” [MS3]	
“I still aim for a productive career but know that having a good support system means more than anything.” [MS3]	

"Work life balance is key" [MS2]	
Concerns about Risk	2
"[…] thinking more about how to care for patients while making sure I don't bring any health concerns (viruses, etc.) back to anyone I'm living with." [MS1]	
"[…] witnessed the potential for harm to physician's families during COVID" [MS2]	
Interest or Greater Interest in Certain Specialties	11
"Taking a global health class during COVID […] solidified my decision to go into med peds" [MS4]	
"COVID-19 caused me to consider more seriously pulm/critical care medicine, as it combines my interest in caring for patients with complex medical problems, procedures, and longitudinal patient care in the outpatient setting." [MS1]	
"COVID-19 has only reinforced my desire to pursue psychiatry. In New York and across the country the prevalence of untreated mental illness among the homeless is particularly problematic in the setting of a global pandemic." [Other]	
"The pandemic highlighted the value of the work of psychiatry for me. While other specialties flagged in terms of demand, psychiatry seemed to become more essential than ever." [MS4]	
Interest in Health Systems	1
"More concerned with opportunity to pursue health equity and systems level change in addition to patient care in light of COVID-19" [MS2]	

Abbreviations: Coronavirus disease 2019 (COVID-19, COVID), Emergency Medicine (EM), Internal Medicine (IM), Emergency Room (ER), Intensive Care Unit (ICU)

[a] Responses from respondents in the primary analysis were counted. The total n does not equal n=212 of the primary analysis as our inclusion criteria did not necessitate participants to provide open-ended responses. Participants could offer multiple reasons.

[b] Responses were counted regardless of participants answering any other parts of the survey. Participants could offer multiple reasons.
